# *Astragalus polysaccharide* alleviates neuropathology and cognitive deficits by modulating gut microbiota and neuroinflammation in an Alzheimer’s disease model

**DOI:** 10.3389/fphar.2026.1830927

**Published:** 2026-08-17

**Authors:** Xiaoli Cui, Zhen Wei, Qingshui Wang, Sizhe Du, Zi Lin, Zibo Chen, Jie Zhang, Chao Li, Lanfang Tang, Xiaoman Dai, Weidong He

**Affiliations:** 1 Department of Geriatric, The People’s Hospital Affiliated to Fujian University of Traditional Chinese Medicine, Fuzhou, China; 2 Department of Neurology, Fuzhou University Affiliated Provincial Hospital, Fujian Provincial Hospital,Shengli Clinical Medical College of Fujian Medical University, Fuzhou, China; 3 The Affiliated People’s Hospital, College of Integrative Medicine, Fujian-Hong Kong-Macau-Taiwan Collaborative Laboratory for the Inheritance and Innovation of Traditional Chinese Medicine, Fujian University of Traditional Chinese Medicine, Fuzhou, China; 4 Department of Neurology and Geriatrics, Fujian Institute of Geriatrics, Fujian Medical University Union Hospital, Fuzhou, China; 5 Fujian Key Laboratory of Molecular Neurology, Fujian Key Laboratory of Vascular Aging, School of Basic Medical Sciences, Fujian Medical University, Fuzhou, China

**Keywords:** Alzheimer’s disease, Astragalus polysaccharides, fecal microbiota transplantation, gut microbiota, microglia, neuroinflammation

## Abstract

**Background:**

Emerging evidence indicates that the neuroprotective effects of *Astragalus polysaccharides* (APS), an extract compound and bioactive constituent derived from traditional Chinese herbs, may be relevant to an effective prescription for delaying progression of Alzheimer’s disease (AD), yet the underlying mechanisms remain to be fully elucidated. This study aimed to investigate the therapeutic efficacy of APS in alleviating cognitive impairment and neuropathology in 5×FAD transgenic mice, with a specific focus on the regulatory role of the gut-brain axis.

**Methods:**

Male 5×FAD mice were orally administered APS (200 mg/kg/day) for 60 days. General observations were conducted to assess the *in vivo* tolerance of APS. Cognitive function was evaluated using the Morris water maze (MWM). Neuropathological assessments included immunofluorescence and Western blotting for amyloid-β (Aβ) deposition, synaptic proteins, and neuroinflammatory markers. Gut microbiota composition and metabolic profiles were analyzed via 16S rRNA gene sequencing and targeted metabolomics. Furthermore, fecal microbiota transplantation (FMT) was performed to verify the causal contribution of gut microbiota to the observed therapeutic effects.

**Results:**

APS administration was well-tolerated throughout the study period, with no overt toxic effects observed. Moreover, APS administration significantly ameliorated spatial learning and memory deficits in 5×FAD mice. Mechanistically, APS treatment reduced Aβ plaque burden, restored synaptic protein expression (PSD-95 and Syntaxin), and attenuated microglia-mediated neuroinflammation by suppressing pro-inflammatory cytokines (IL-6, TNF-α) and upregulating TREM2. Microbiome analysis revealed that APS reshaped gut microbial diversity and composition, enriching beneficial taxa such as *Lactobacillus*. Metabolomics indicated a partial restoration of amino acid metabolism. Notably, FMT from APS-treated donors successfully reproduced the cognitive improvements and anti-inflammatory effects in recipient mice.

**Conclusion:**

These findings demonstrate that APS alleviates cognitive deficits and AD-like pathology, partially through remodeling gut microbiota and modulating the gut-brain axis. APS represents a promising natural compound-based therapeutic candidate for managing cognitive decline associated with Alzheimer’s disease.

## 1 Introduction

Alzheimer’s disease (AD) is a progressive neurodegenerative disorder characterized by cognitive decline and memory impairment, which is marked by the accumulation of amyloid-β plaques and neurofibrillary tangles, leading to synaptic dysfunction, neuroinflammation and neuronal loss (Jia et al., 2020; Kamatham et al., 2024). Both traditional anti-dementia drugs, such as cholinesterase inhibitors (e.g., donepezil) and N-methyl-D-aspartate (NMDA) receptor antagonists (e.g., memantine), which mainly provide symptomatic relief, and novel disease-modifying therapies such as lecanemab and donanemab still have important limitations (Salloway et al., 2025; Sims et al., 2023; van Dyck et al., 2023; Veroniki et al., 2022). The inflammatory response mediated by microglia in the central nervous system (CNS) is critically involved in AD pathology (Ising et al., 2019). Specifically, abnormal Aβ and Tau proteins drive microglial overactivation, which secretes various inflammatory mediators (e.g., IL-1β, TNF-α, IL-6) that exert neurotoxic effects and lead to neuronal and synaptic dysfunction (Chinnathambi et al., 2025). Therefore, inhibiting this neuroinflammatory response has recently emerged as an important therapeutic direction for AD (Lucena and Heneka, 2024).

With compelling evidence implicating the gut microbiota in AD progression, the gut-brain axis has emerged as a critical pathway in the pathogenesis of Alzheimer’s disease. Significant dysbiosis, characterized by altered microbial diversity and composition, is a consistent feature in AD patients and animal models (Abdol Samat et al., 2025). This imbalance is intimately linked to heightened neuroinflammation, a central driver of synaptic dysfunction and neuronal loss, which ultimately exacerbates cognitive decline (Zhang et al., 2025). Plasma and fecal multi-omics studies have revealed that the severity of cognitive impairment in Alzheimer’s disease is closely associated with reduced alpha-diversity of the gut microbiota, proliferation of pro-inflammatory bacteria (such as Proteobacteria), and accompanying systemic inflammatory responses and alterations in the metabolite profile (Hosseinkhani et al., 2025). Increasing evidence has demonstrated that modulating the perturbed gut microbiota can attenuate neuroinflammatory responses and ameliorate cognitive deficits, highlighting microbial-targeted strategies as a promising therapeutic Frontier for AD (Kapoor et al., 2024; Wang et al., 2019). In this context, natural compounds with prebiotic potential offer a viable approach.

*Astragalus polysaccharide* (APS), a water-soluble heteropolysaccharide complex primarily composed of neutral monosaccharides (with glucose as the dominant component, along with trace amounts of arabinose, galactose, mannose, rhamnose, and galacturonic acid), was extracted from the traditional Chinese medicinal herb *Astragalus membranaceus* (Fu et al., 2014; Chen et al., 2023). APS has demonstrated a wide range of pharmacological activities, such as antioxidant, immunomodulatory, anti-inflammatory, and neuroprotective effects in critical brain processes such as acute ischemic stroke (Li et al., 2025; Shi and Ma, 2024), experimental autoimmune encephalomyelitis (Ma et al., 2024) and neurodegenerative disease models, mostly through regulating microglial function (Shi and Ma, 2024). Preliminary studies suggest that APS ameliorate obesity and insulin resistance, improves cognition-related nesting behavior and suppresses the activation of plaque-associated microglia in the APP/PS1 mice subjected to metabolic stress induced by a high-fat diet combined with low-dose streptozotocin (Huang et al., 2017), while exerting no significant effects on amyloid-β (Aβ) deposition or serum Aβ levels. Although APS alleviates cognitive impairment and amyloid accumulation via regulating oxidative stress in AD mice (Qin et al., 2020), the precise molecular mechanisms by which APS rectifies gut dysbiosis and subsequently mitigate neuroinflammation in AD remain to be fully elucidated.

Furthermore, elevated production of short-chain fatty acids (SCFAs), such as butyric acid (BA) and iso-butyric acid (IBA), by the gut microbiota has been reported to correlate positively with cerebral Aβ deposition and proinflammatory factors (Gu et al., 2024). However, it remains unclear whether APS can ameliorate AD-related pathologies by remodeling the gut microbial structure and subsequently regulating microbial metabolites, including amino acids and neurotransmitters. Increasing evidence has demonstrated that fecal microbiota transplantation (FMT) exerts therapeutic effects by modulating the gut-brain axis (Upadhyay et al., 2025). Transplantation of fecal microbiota from healthy donor mice into AD model recipients resulted in significant improvements in spatial memory and exploratory behaviors, a reduction in β-amyloid plaques in the brain, a decrease in the levels of inflammatory factors, and an increase in neurotransmitters such as acetylcholine. Conversely, susceptible mice receiving the patient colony showed accelerated cognitive decline and exacerbated pathological features in 5×FAD models (Benichou Haziot and Birak, 2023; Elangovan et al., 2022; Kim et al., 2021).

To bridge the knowledge gap, this study aims to investigate the therapeutic potential of APS in cognitive impairment in 5×FAD transgenic mice, employing both oral administration and FMT approaches. Meanwhile, we assessed the impact of APS on amyloid-β plaque deposition, synaptic protein expression. Furthermore, 16S rRNA gene sequencing and metabolomics analyses will be integrated to delineate the associated alterations in gut microbiota composition and metabolic activity. To this end, neuroinflammatory cytokines such as the interleukin-1β (IL-1β), interleukin-6 (IL-6), and tumor necrosis factor-α (TNF-α), transforming growth factor-beta 1 (TGF-β1) and triggering receptor expressed on myeloid cells 2 (TREM2) expression were quantified using commercial ELISA kits which represent characteristics of M1/M2 microglia polarization states in the brain (Darwish et al., 2023).

## 2 Materials and methods

### 2.1 Drugs and reagents

APS was purchased from Meryer Chemical Technology Co., Ltd. (Shanghai, China). Prior to administration, APS was dissolved in sterile physiological saline to achieve the desired concentration. The human APP695-specific DNA primers (follows: Forward: 5′-AGA​GTA​CCA​ACT​TGC​ATG​ACT​ACG-3′; Reverse: 5′-ATG​CTG​GAT​AAC​TGC​CTT​CTT​ATC-3′) for genotyping 5×FAD transgenic mice were synthesized by Sangon Biotech (Shanghai, China). Genotyping was performed using a standard polymerase chain reaction (PCR) protocol to detect the expression of the human APP transgene (Zhang and Zhang, 2025).

### 2.2 Experimental Animals and grouping

Male 5×FAD transgenic mice (C57BL/6J background, 4-month-old) and wild-type littermates were used in this study. The 5×FAD mouse colony was originally established and stably maintained at Fujian Medical University, with founder breeding pairs sourced from The Jackson Laboratory (Stock No. 034848-JAX; Bar Harbor, ME, USA). These mice carry five familial Alzheimer’s disease (FAD) mutations driven by the neuron-specific murine Thy-1 promoter, leading to abundant Aβ42 production: three APP mutations (K670N/M671L [Swedish], I716V [Florida], V717I [London]) and two PSEN1 mutations (M146L, L286V) (Oakley et al., 2006). Mice were housed at three to five per cage under a 12 h light/dark cycle with *ad libitum* access to standard chow and autoclaved water, and were acclimatized to the facility environment for at least 1 week prior to any experimental manipulation.

For the first cohort, 5×FAD mice were randomly divided into an APS-treated group (n = 8–10, 200 mg/kg/day, oral gavage, 60 consecutive days) or a vehicle control group (n = 8, equal volume of normal saline). Fecal samples were collected post-treatment, followed by behavioral assessments. An additional cohort was assigned to three FMT groups, n = 8 for each group: (1) FMT-5×FAD (donor: untreated 5×FAD), (2) FMT-vehicle (donor: saline-treated 5×FAD), and (3) FMT-5×FAD + APS (donor: APS-treated 5×FAD). Fresh fecal samples which were collected from each donor mouse of the first cohort (APS-treated or saline-treated were homogenized in 0.9% NaCl (1 g: 10 mL) for 5 min, supplemented with 10% glycerol (0.1 mL), and stored. For FMT, recipient mice were orally gavaged with an antibiotic cocktail containing metronidazole 1 g/L, vancomycin 1 g/L and streptomycin 2 g/L for 3 consecutive days once only, prior to the initial transplantation,no additional antibiotics were given before subsequent weekly transfers. To ensure a 1:1 donor-torecipient ratio, each recipient mouse was paired with a specific donor from the corresponding treatment group. The entire fecal suspension derived from one donor was orally gavaged to its paired recipient at a dose of 1 mL per 100 g body weight. Recipient mice received this individually matched suspension once weekly for 8 consecutive weeks. All procedures were approved by the Institutional Animal Care and Use Committee of Fujian Medical University (FJMU ACUC 2021-0399) and performed in accordance with the European Union Guidelines for the Care and Use of Experimental Animals (Directive 2010/63/EU).

### 2.3 Behavioral testing

Spatial learning and memory were assessed using the Morris water maze (MWM) test (Model: MWM-1000, Nanjing Kalvin Experimental Equipment Co., Ltd., China). The apparatus consisted of a circular pool (120 cm in diameter, 50 cm in height), a removable hidden platform (10 cm in diameter), and a video tracking system (version 2.0). During the hidden platform training phase, mice underwent four trials per day for 5 consecutive days. The escape latency (time taken to locate the hidden platform, with a maximum of 60 s) was recorded for each trial. If a mouse failed to find the platform within 60 s, it was gently guided to the platform and allowed to remain there for 15 s. The platform location remained constant across trials, while the starting positions were varied randomly. A probe trial was conducted 24 h after the last training session to evaluate memory retention. The platform was removed, and each mouse was allowed to swim freely for 60 s. The swimming trajectory, number of crossings over the former platform location, time spent in the target quadrant and escape latency were recorded and analyzed.

### 2.4 Fecal sample collection

As previously described (Zhao et al., 2021), at the end of the treatment period, fresh fecal pellets were collected from all mice and immediately frozen at −80 °C for subsequent 16S rRNA gene sequencing and targeted metabolomic analysis.

### 2.5 Tissue harvesting

After behavioral testing, mice were anesthetized and euthanized. The brain was rapidly dissected and divided sagittally: the right hemisphere was fixed in 4% paraformaldehyde for 24 h, dehydrated, flash-frozen, and stored at −80 °C until sectioning. The left hemisphere was microdissected to isolate the hippocampus and cortex, which were snap-frozen in liquid nitrogen and stored at −80 °C for biochemical analyses.

### 2.6 Immunofluorescence staining

Fixed brain tissue specimens were dehydrated through a graded ethanol series, cleared with xylene, embedded in paraffin, and sectioned. Sections were deparaffinized in xylene and rehydrated through a graded ethanol series. After thawing and washing with PBS, sections were permeabilized with 0.5% Triton X-100 (in pBS) for 10 min at room temperature. After blocking with 5% normal serum for 30 min at 37 °C, sections were incubated with primary antibodies: mouse anti-6E10 (clone 6E10, recognizing the 1–16 amino acid sequence of β-Amyloid; 1:200, Biolegend, #SIG-39320) and Rabbit Anti-Iba1 (1:100, proteintech, #10904-1-AP) at 4 °C overnight. The following day, sections were washed and incubated with species-appropriate fluorescent secondary antibodies Cy3-conjugated goat anti-mouse IgG (1:200, ABclonal, # A5008) or Alexa Fluor 488-conjugated goat anti-rabbit IgG (1:200, ZSGB-BIO, # ZF-0511) for 45 min at 37 °C. Nuclei were counterstained with DAPI. All steps involving fluorophores were performed in the dark. Images were acquired using a Zeiss Imager. M2 fluorescence microscope with appropriate filter sets: DAPI (excitation 330–380 nm, emission 420 nm), FITC (excitation 465–495 nm, emission 515–555 nm), and Cy3 (excitation 510–560 nm, emission 590 nm). Quantification of Iba-1 immunofluorescence intensity was carried out using Zeiss ZEN software (blue edition, Carl Zeiss Microscopy). For each brain section, at least three fields of view were randomly selected and captured under identical acquisition settings. The mean fluorescence intensity of Iba-1-positive and Aβ areas was measured after background subtraction. The relative fluorescence intensity per animal was normalized to the average intensity of the WT control group. Data from six animals per group were pooled and presented as mean ± SEM.”

### 2.7 Western blot analysis

Hippocampal tissues were harvested and homogenized in pre-chilled tissue protein lysis buffer. The lysis buffer consisted of pH7.4 TBS supplemented with 1% Triton X-100, 50 mM sodium fluoride, 2 mM sodium orthovanadate, 10 mM sodium pyrophosphate, and 1% protease inhibitor cocktail. Tissue samples were further lysed by ultrasonic disruption using a JY92-IIN ultrasonic homogenizer (Ningbo Xinzhi Biotechnology Co., Ltd., China). Protein concentration was determined using a BCA assay kit (Beyotime, China). Equal amounts of protein were separated by 10% SDS-PAGE and transferred to PVDF membranes. After blocking with 5% non-fat milk for 2 h, membranes were incubated with primary antibodies:Mouse Anti-GAPDH (1:2000,TransGen Biotech,#HC301), Rabbit Anti PSD-95 (1:1000,Affinity Biosciences, #AF5283), Rabbit Anti Syntaxin (1:1000, Abclonal,#A19813), Rabbit Anti IBA-1 (1:5000,proteintech,#81728-1-RR) at 4 °C overnight. Following TBST washes, membranes were incubated with HRP-conjugated secondary antibodies:Goat Anti-Rabbit IgG (1:2000) for 2 h at room temperature. Protein bands were visualized using an enhanced chemiluminescence (ECL) detection system on a high-sensitivity chemiluminescence imaging instrument.

### 2.8 ELISA for inflammatory cytokines

Hippocampal homogenates were prepared as described above. Levels of inflammatory cytokines (TNF-α, IL-6,TREM2 and TGF-β1) were quantified using commercial ELISA kits according to the manufacturer’s instructions. Specifically, the Mouse IL-6 ELISA Kit (Catalog No. E-MSEL-M0001, Elabscience Biotechnology Co., Ltd., China), Mouse TNF-α ELISA Kit (Catalog No. E-MSEL-M0002, Elabscience Biotechnology Co., Ltd., China), and Mouse TGF-β1 ELISA Kit (Catalog No. E-EL-R0162, Elabscience Biotechnology Co., Ltd., China) were purchased from Elabscience. The Mouse TREM2 ELISA Kit (Catalog No. MM-51213M2, Jiangsu Meimian Industrial Co., Ltd., China) was obtained from Jiangsu Meimian. All optical density (OD) values were measured using a microplate reader, and the concentrations of target proteins were calculated based on the standard curve.

### 2.9 Microbiota analysis

To comprehensively characterize the gut microbial community and its functional potential, fecal DNA was subjected to 16S rRNA gene sequencing. Raw sequencing reads were processed using fastp (v0.23.4) for quality filtering and FLASH (v1.2.11) for merging paired-end reads. Clean sequences were then denoised into amplicon sequence variants (ASVs) using the Deblur algorithm within QIIME2 (v2024.5). Taxonomic assignment of ASVs was performed against the SILVA database (v138.1). Alpha diversity (within-sample richness and evenness) was evaluated using indices including Chao1, while beta diversity (between-sample community structure differences) was assessed and visualized via dimensionality-reduction techniques such as principal coordinate analysis (PCoA), principal component analysis (PCA), and non-metric multidimensional scaling (NMDS).

To predict the functional gene content of the microbial communities, Phylogenetic Investigation of Communities by Reconstruction of Unobserved States 2 (PICRUSt2, v2.5.3) was applied to the 16S rRNA gene sequencing data. This enabled the inference of Kyoto Encyclopedia of Genes and Genomes (KEGG) orthologs and subsequent pathway-level functional profiles. Differential KEGG pathway enrichment analysis between experimental groups was performed using the metagenomeSeq R package. The specific pairwise comparisons conducted were: (1) wild-type (WT) vs. 5×FAD mice (5×FAD); (2) WT vs. APS-treated 5×FAD mice (5×FAD + APS); and (3) 5×FAD vs. 5×FAD + APS. Pathways with a corrected P-value <0.05 (Benjamini–Hochberg false discovery rate correction) were considered statistically significant.

### 2.10 16S rRNA gene sequencing and library construction

Fecal genomic DNA was extracted using the TIANamp Stool DNA Kit (Cat# DP328, Tiangen Biotech, Beijing, China) according to the manufacturer’s instructions with minor modifications. Briefly, 180–220 mg of stool sample was mixed with 500 μL Buffer SA, 100 μL Buffer SC, 15 μL Proteinase K, and 0.25 g of grinding beads. The mixture was incubated at 70 °C for 15 min, followed by centrifugation at 12,000×g for 3 min. The supernatant was treated with 10 μL RNase A for 5 min at room temperature. After adding 200 μL Buffer SH and incubating on ice for 5 min, the sample was centrifuged, and the resulting supernatant was mixed with an equal volume of Buffer GFA. The mixture was then loaded onto a CR2 spin column. The column was washed sequentially with 500 μL Buffer GD and twice with 700 μL Buffer PW, each with a centrifugation step at 12,000×g for 30 s. A final dry centrifugation at 12,000×g for 2 min was performed to remove residual ethanol. DNA was eluted with 50 μL Buffer TB by incubating for 2–5 min at room temperature, followed by centrifugation at 12,000×g for 2 min. Purified DNA was stored at −20 °C, and its purity was verified by measuring the OD260/OD280 ratio (acceptable range: 1.7–1.9). Following quality control, sequencing libraries were constructed and subjected to paired-end sequencing on the Illumina NovaSeq 6000 platform.

### 2.11 LC/MS-based targeted amino acid metabolomics analysis

A targeted metabolomics approach was employed to quantitatively profile alterations in amino acid metabolism, measuring over 90 amino acids and related metabolites. For sample preparation, fecal samples from Alzheimer’s disease model mice were homogenized and extracted with 80% cold methanol (v/v) to precipitate proteins. After centrifugation (12,000×g, 15 min, 4 °C), the supernatant was collected, vacuum-dried, and reconstituted in 50% methanol prior to liquid chromatography-tandem mass spectrometry (LC-MS/MS) analysis. Mass spectrometric detection was carried out using a triple quadrupole instrument operating in multiple reaction monitoring (MRM) mode with electrospray ionization in both positive and negative modes. For data processing and quality control, raw data were processed to extract and align peaks, calibrate retention times, and plot total ion chromatograms (TIC). System stability and data reproducibility were evaluated using quality control (QC) samples. Metabolites were annotated by matching accurate mass and fragmentation spectra against the MWDB database. Only metabolites with a relative standard deviation (RSD) < 15% in QC samples were retained for subsequent analysis. Differentially abundant metabolites between groups were identified based on multivariate statistical methods and visualized using clustering heatmaps.

### 2.12 Statistical analysis

All data are expressed as mean ± standard error of the mean (SEM). Statistical analyses were performed using Graphpad prism software (version 9.0). Differences between groups were assessed using one-way or two-way analysis of variance (ANOVA) followed by appropriate *post hoc* tests. A p-value of less than 0.05 was considered statistically significant.

## 3 Result

### 3.1 APS improved cognitive function in 5×FAD mice

**FIGURE 1:**
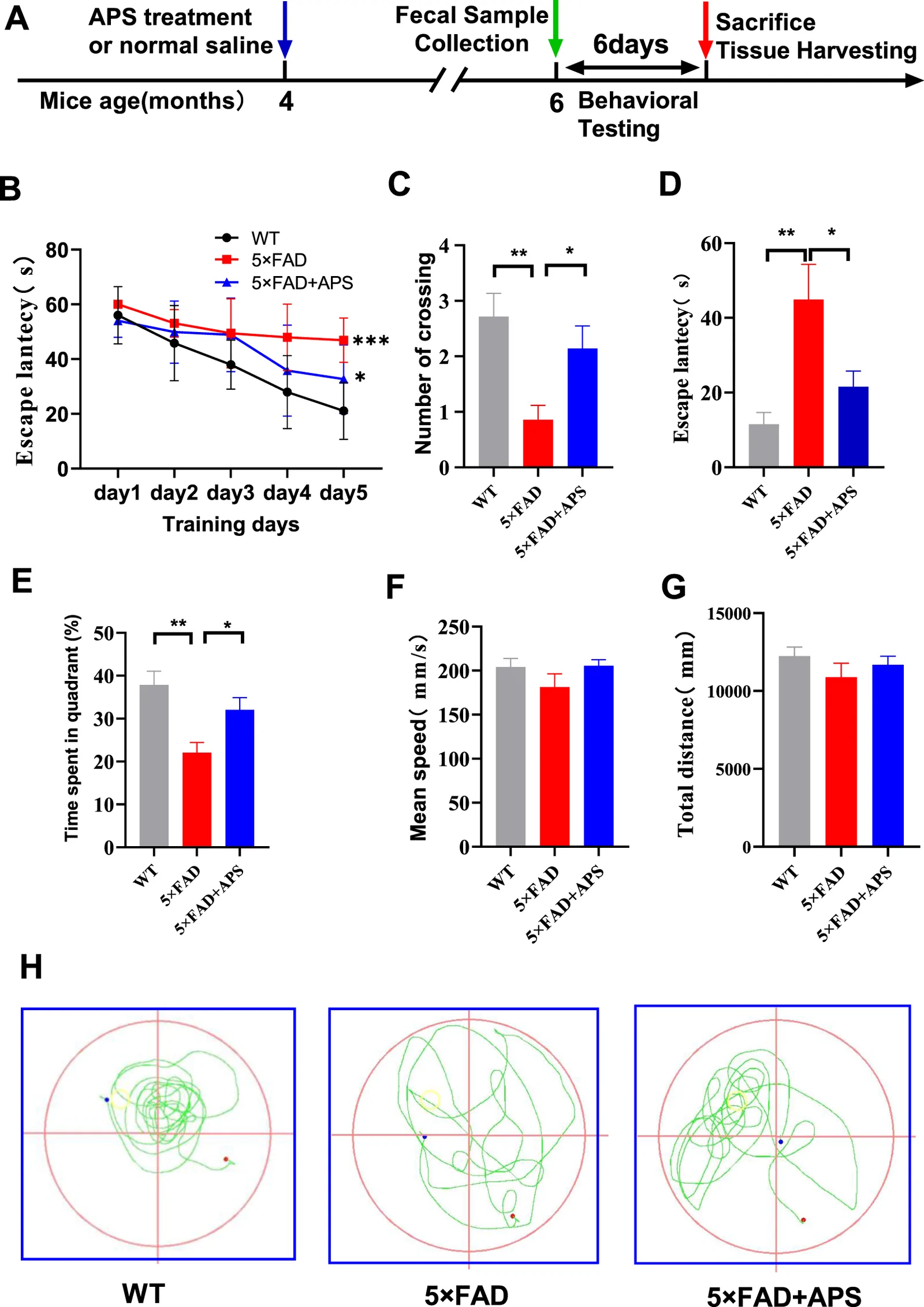
Behavioral effects of APS treatment in 5×FAD mice. **(A)** Experimental timeline: APS or normal saline treatment, fecal sample collection, behavioral tests and tissue harvesting. **(B–H)** Behavioral performance in Morris water maze tests: **(B)** Escape latency during 5-day Morris water maze training (***p < 0.001, 5×FAD vs*.* WT, *p < 0.05, 5×FAD + APS vs*.* 5×FAD); Figures C–H display the probe trial data on day 6: **(C) **Number of crossing; **(D)** Escape latency; **(E)** Time spent in target quadrant; **(F)** Mean swimming speed; **(G)** Total swimming distance; **(H)** Movement trajectories. Data are presented as mean ± SEM; n = 8–10. *p < 0.05, **p < 0.01,***p < 0.001.

The general experimental design and sampling timeline are displayed in Figure 1A. To assess the *in vivo* safety of APS, hematological parameters and liver and kidney function indicators were examined after the treatment period. No significant differences were observed among the experimental groups in white blood cell counts, red blood cell counts, platelet counts, as well as levels of alanine aminotransferase, aspartate aminotransferase, and creatinine (all p > 0.05). These findings indicate that, under the present experimental conditions, APS did not induce apparent hematological toxicity or hepatorenal injury, suggesting a favorable *in vivo* safety profile (data are not shown in the main text).

The Morris water maze test was employed to evaluate the effects of APS on cognitive function in 5×FAD mice. During the training phase, 5×FAD mice exhibited significantly longer escape latencies compared to wild-type (WT) controls (*p* < 0.001). APS treatment progressively shortened the escape latency over the training days (*p* < 0.05) (Figure 1B). In the probe trial conducted after 5 days of training, the swimming trajectories of 5×FAD mice appeared disorganized with limited exploration in the target quadrant, whereas APS-treated mice showed more focused swimming patterns in the platform-containing quadrant (Figure 1E). Quantitative analysis revealed no significant differences in swimming speed and total distance among groups (Figures 1F,G) (*p* > 0.05). Compared to WT mice, 5×FAD mice demonstrated prolonged escape latency (Figure 1D) (*p* < 0.01), reduced platform crossings (Figure 1C) (*p* < 0.01), and decreased time spent in the target quadrant (Figure 1E) (*p* < 0.01). APS treatment significantly reversed these deficits, as evidenced by shortened escape latency (*p* < 0.05), increased platform crossings (*p* < 0.05), and extended target quadrant duration (*p* < 0.05) compared to untreated 5×FAD mice. Meanwhile, the swimming movement trajectories revealed that WT mice displayed concentrated and active exploratory behavior, whereas 5×FAD mice presented restricted and fragmented locomotion (Figure 1H). Notably, mice in the astragalus polysaccharide group mostly stayed in the quadrant where the platform was located during swimming.

### 3.2 APS attenuated microglial activation and neuroinflammation in 5×FAD mice

**FIGURE 2:**
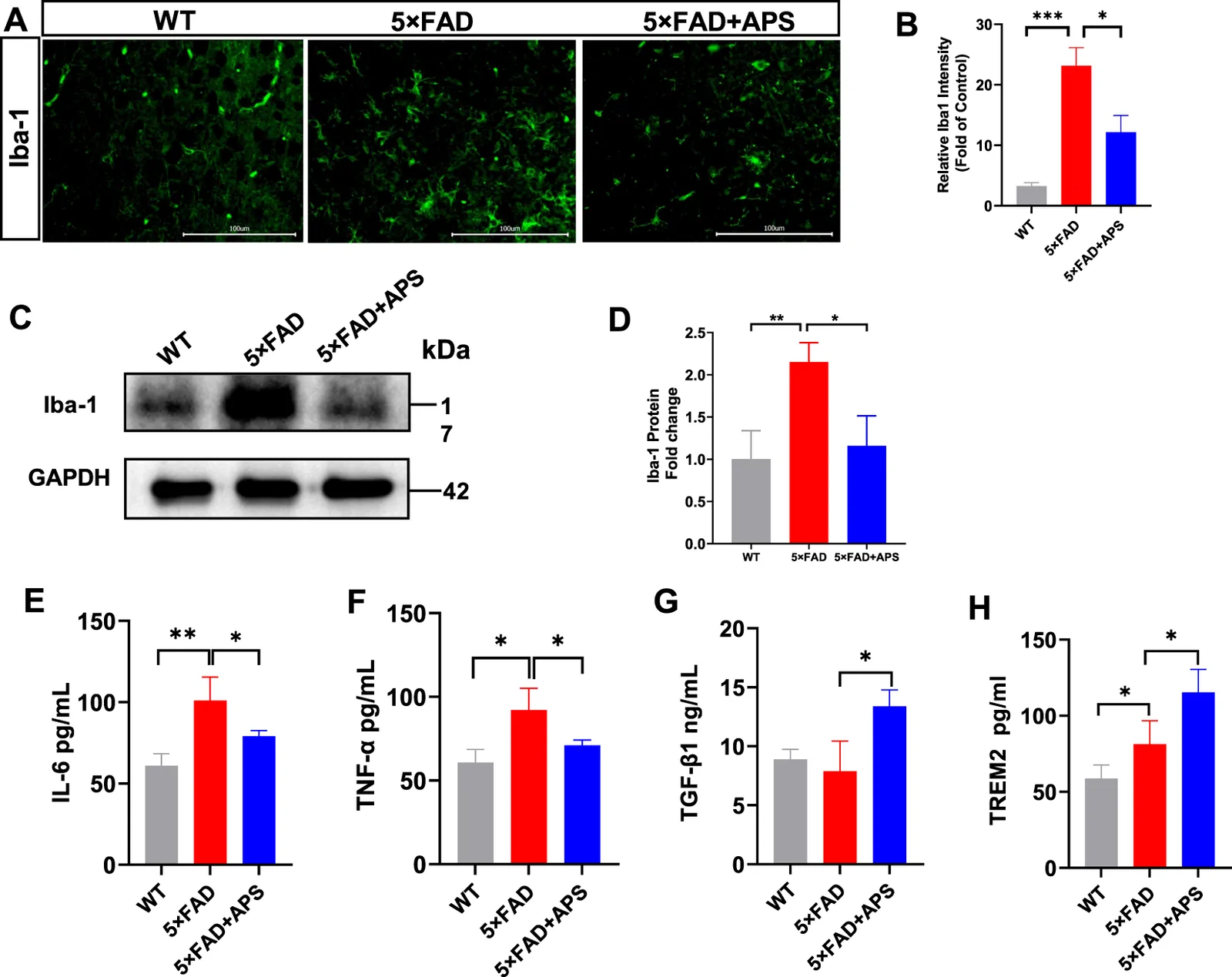
APS attenuates microglial activation and inflammatory response in 5×FAD mice. **(A,B)** Microglial activation assessed by Iba-1 immunofluorescence staining. **(A)** Representative immunofluorescence images of Iba-1 (microglial marker, green fluorescence) in brain sections. Scale bar = 100 μm. **(B)** Quantitative analysis of relative Iba-1 fluorescence intensity (normalized to WT group). **(C,D)** Iba-1 protein expression detected by Western blotting: **(C)** Representative Western blot bands of Iba-1 and GAPDH in brain tissues of each group. **(D)** Quantitative analysis of relative Iba-1 protein level (normalized to GAPDH, with WT group as control). **(E–H)** Quantification of inflammatory cytokines and microglial-related molecules Concentrations of **(E)** IL-6, **(F)** TNF-α, **(G)** TGF-β1, and **(H)** TREM2 in brain tissues were measured by ELISA. Data are presented as mean ± SEM (n = 3 in A-D; n = 4 in **(E–H)**; *p < 0.05, **p < 0.01, ***p < 0.001.

To evaluate the effect of APS on microglial activation in 5×FAD mice, we first performed Iba-1 immunofluorescence staining. As shown in Figures 2A,B, the immunofluorescence staining of the microglial marker Iba1 (Ionized calcium-binding adapter molecule 1, a protein specifically expressed in microglia/macrophages that is upregulated during their activation and used to assess microglial activation status) revealed prominent microglial activation in 5×FAD mice, characterized by hypertrophic cell bodies with thickened and shortened processes, and clustered distribution patterns. In contrast, WT mice displayed resting microglia with small cell bodies and fine, elongated processes. APS treatment reduced the number of activated microglia while increasing the population of resting microglia. Quantitative analysis of immunofluorescence (*p* < 0.001, Figure 2B) and Western blot (*p* < 0.01, Figures 2C,D), confirmed significantly elevated Iba1 expression in 5×FAD mice, which were effectively reduced by APS treatment (*p* < 0.05, Figures 2B,D).

To delineate the impact of APS on neuroinflammation in 5×FAD mice, we quantified the levels of key inflammatory mediators in brain tissues via ELISA. As presented in Figures 2E,F, the concentrations of pro-inflammatory cytokines IL-6 and TNF-α were significantly elevated in the 5×FAD group (*p* < 0.01, *p* < 0.05 vs. WT group, respectively), while APS treatment significantly reduced their levels (*p* < 0.05 vs. 5×FAD group). For the anti-inflammatory cytokine TGF-β1 (Figure 2G) and microglial phagocytosis-related molecule TREM2 (Figure 2H), the 5×FAD group showed a slight increase (TREM2: *p* < 0.05 vs. WT group), and APS further promoted the expressions of TGF-β1 and TREM2 (*p* < 0.05 vs. 5×FAD group for TGF-β1 and TREM2). Collectively, these data demonstrate that APS suppresses microglial overactivation and modulates the inflammatory microenvironment in 5×FAD mice.

### 3.3 APS ameliorated neuropathology and promoted synaptic protein expression

**FIGURE 3:**
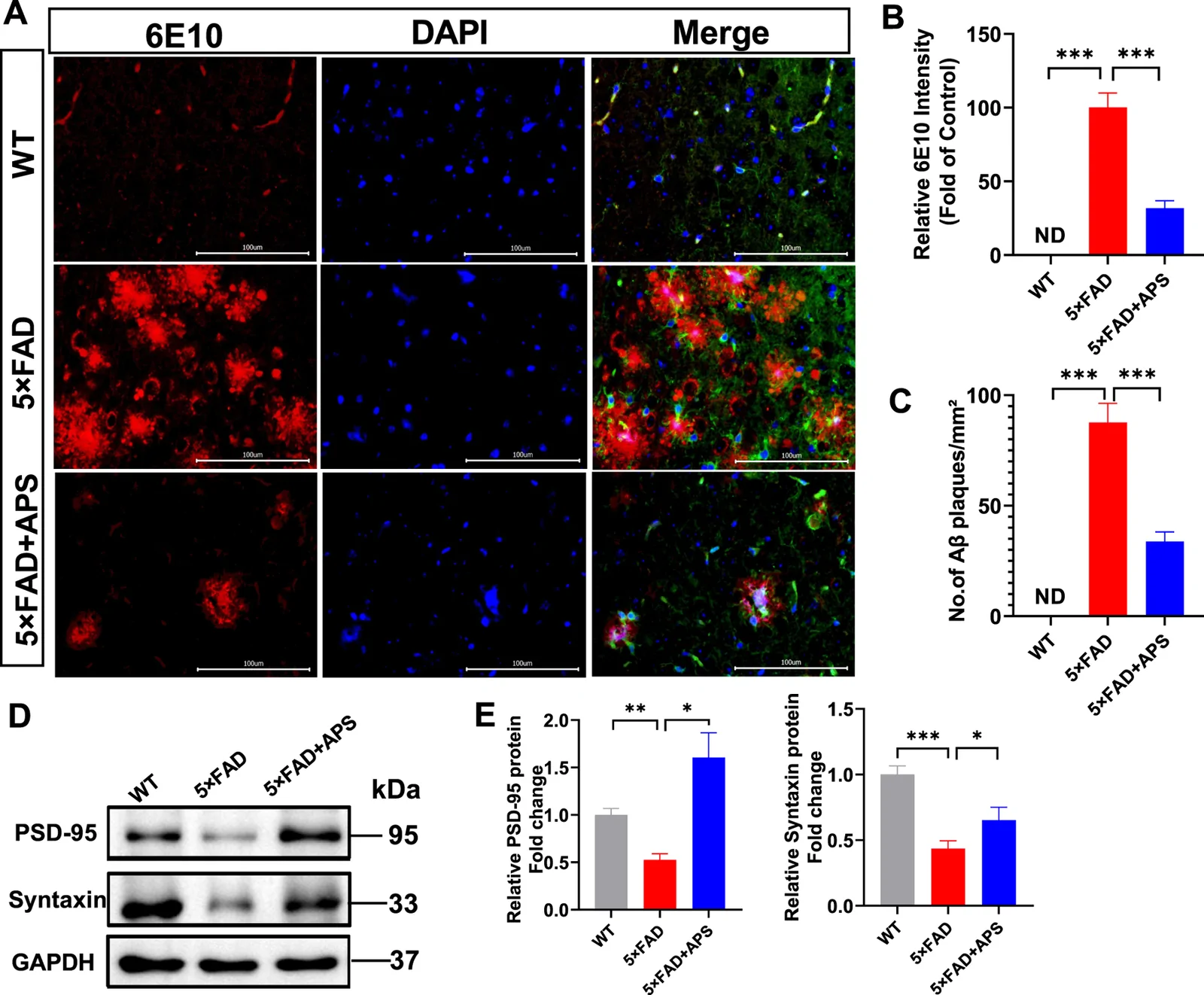
APS reduces Aβ deposition and rescues synaptic protein loss in 5×FAD mice. **(A)** Representative immunofluorescence images showing Aβ plaques stained with anti-Aβ antibody (red) and nuclear staining (DAPI, blue) in the cortex of WT, 5×FAD, and 5×FAD + APS mice (scale bar: 100 μm). **(B)** Quantitative analysis of relative Aβ fluorescence intensity. **(C)** Quantification of Aβ plaque number per mm^2^ in cortical sections. **(D)** Representative Western blot bands of synaptic proteins (PSD-95, Syntaxin) and the internal control GAPDH in brain tissues. **(E)** Densitometric analysis of PSD-95 and Syntaxin protein levels. Data are presented as mean ± SEM (n = 3 in **(A–D)**. ND: not detected; *p < 0.05, **p < 0.01, ***p < 0.001.

To assess the effect of APS on Aβ pathology in 5×FAD mice, we first examined cortical Aβ deposition via immunofluorescence. As shown in Figure 3A, Aβ plaques (red signals) were undetectable in WT mice, while robust Aβ aggregation was observed in 5×FAD mice. Notably, APS treatment significantly reduced Aβ fluorescence intensity (Figure 3B) and plaque density (Figure 3C) in 5×FAD mice (both *p* < 0.001 vs. 5×FAD). Synaptic loss is a hallmark of cognitive decline in AD. We thus measured synaptic proteins via Western blotting. 5×FAD mice exhibited marked reductions in PSD-95 and Syntaxin levels relative to WT controls (*p* < 0.01 and *p* < 0.001), whereas APS administration partially rescued the downregulation of these proteins (Figures 3D,E; *p* < 0.05 vs. 5×FAD). These data suggest that APS mitigates Aβ pathology and preserves synaptic integrity in 5×FAD mice.

### 3.4 APS modulated gut microbiota composition in 5×FAD mice

**FIGURE 4:**
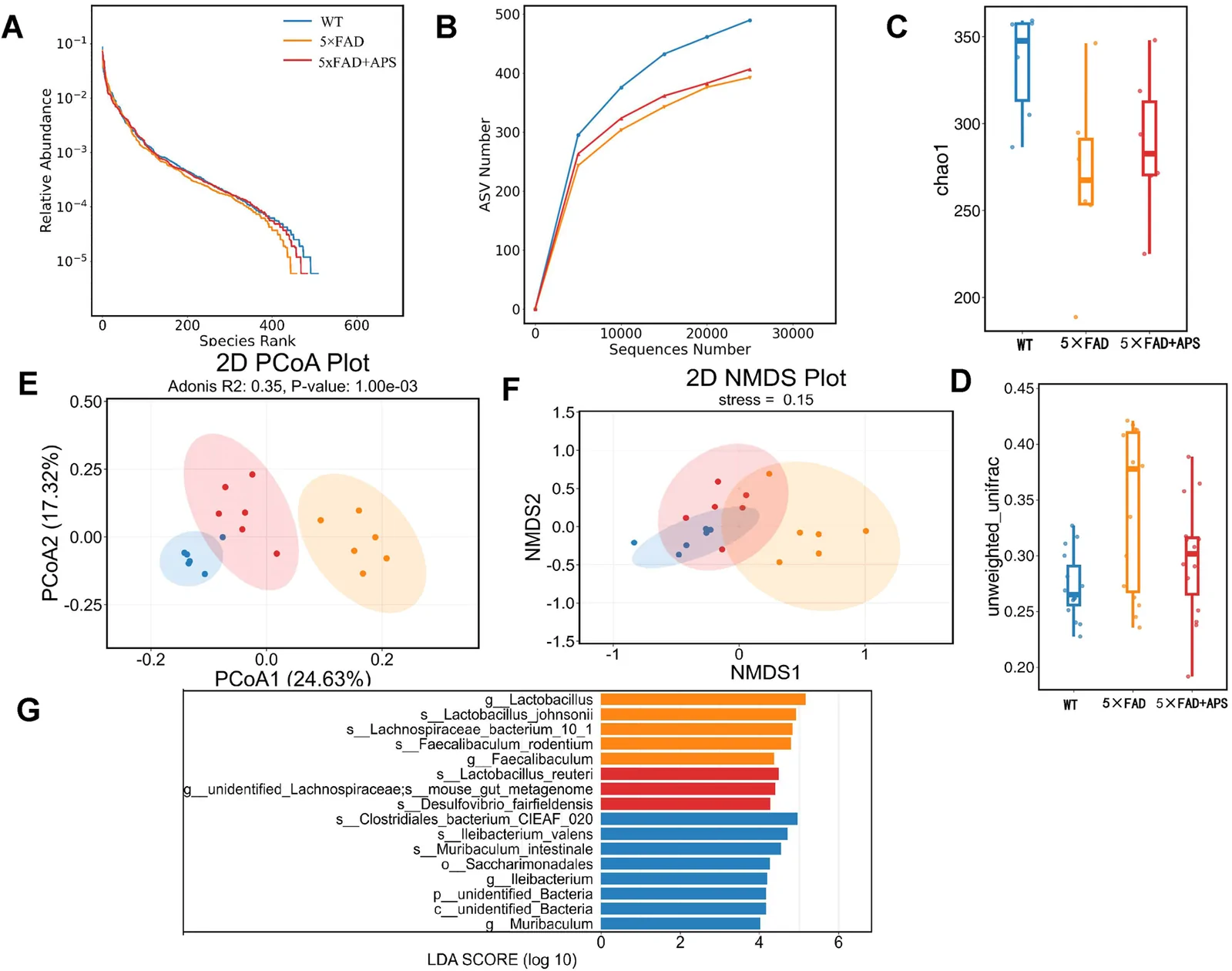
Effects of APS intervention on gut microbial diversity and composition in 5×FAD mice. **(A)** Species rank abundance curve, showing microbial species richness and evenness across groups. **(B)** Rarefaction curve, demonstrating that sequencing depth was sufficient to capture the majority of species in all groups. **(C)** Boxplot of observed amplicon sequence variants (ASVs), representing the number of unique microbial species detected per sample (a measure of alpha-diversity). **(D)** Boxplot of unweighted UniFrac distances, reflecting intra-group heterogeneity in microbial community composition. **(E)** Principal coordinate analysis (PCoA) plot based on unweighted UniFrac distances, showing distinct clustering of microbial communities among groups (Adonis *R* ^2^ = 0.35, *p* = 0.001). **(F)** Non-metric multidimensional scaling (NMDS) plot, further confirming significant structural differences in gut microbiota (stress = 0.15, indicating reliable ordination). **(G)** Linear discriminant analysis effect size (LEfSe) identifying differentially abundant microbial taxa across groups:The bar plot displays microbial taxa with significant differential abundance (LDA score >4) among wild-type control, 5×FAD and 5×FAD + ApS groups. LDA score (log_10_) indicates the magnitude of the effect size for each taxon in distinguishing groups. (n = 6 for each group).

We next characterized gut microbiota differences among WT, 5×FAD, and APS-treated 5×FAD mice via 16S rRNA gene sequencing; rank-abundance (Figure 4A) and rarefaction curves (Figure 4B) indicated adequate coverage across groups. Alpha diversity analysis showed that 5×FAD mice exhibited reduced microbial richness and diversity relative to WT controls (Chao1 index, Figure 4C; unweighted UniFrac; Figure 4D), indicating baseline dysbiosis in the AD model. PCoA based on unweighted UniFrac distances (Figure 4E) showed clear separation between WT and 5×FAD communities (Adonis R^2^ = 0.35, p = 0.001), and NMDS analysis yielded consistent results (Figure 4F, stress = 0.15), confirming marked structural differences at baseline.

Building on these baseline differences, APS treatment partially restored alpha diversity-diversity indices and reduced intra-group heterogeneity (Figures 4C,D), and shifted the 5×FAD microbial community toward the WT profile in ordination space (Figures 4E,F). These results indicate that APS ameliorates AD-associated dysbiosis at both diversity and community-structure levels.

Linear discriminant analysis effect size (LEfSe) identified key taxa associated with group differences (Figure 4G, LDA score >4). At the genus level, 5×FAD mice were enriched in *Lactobacillus* and Faecalibaculum, whereas WT microbiota were dominated by core commensals such as Muribaculum and Ileibacterium. Following APS intervention, 5×FAD + APS mice were enriched in *Lactobacillus* reuteri and an unidentified Lachnospiraceae genus, taxa with reported barrier-supporting (Gao et al., 2022) and putative butyrate-producing properties that are relevant to neuroinflammation control (Wang et al., 2023). Together, these findings suggest that APS not only improves overall diversity and structure but also selectively enriches taxa with neuroprotective potential.

### 3.5 APS reshapes gut microbial functional pathways in 5×FAD mice

**FIGURE 5:**
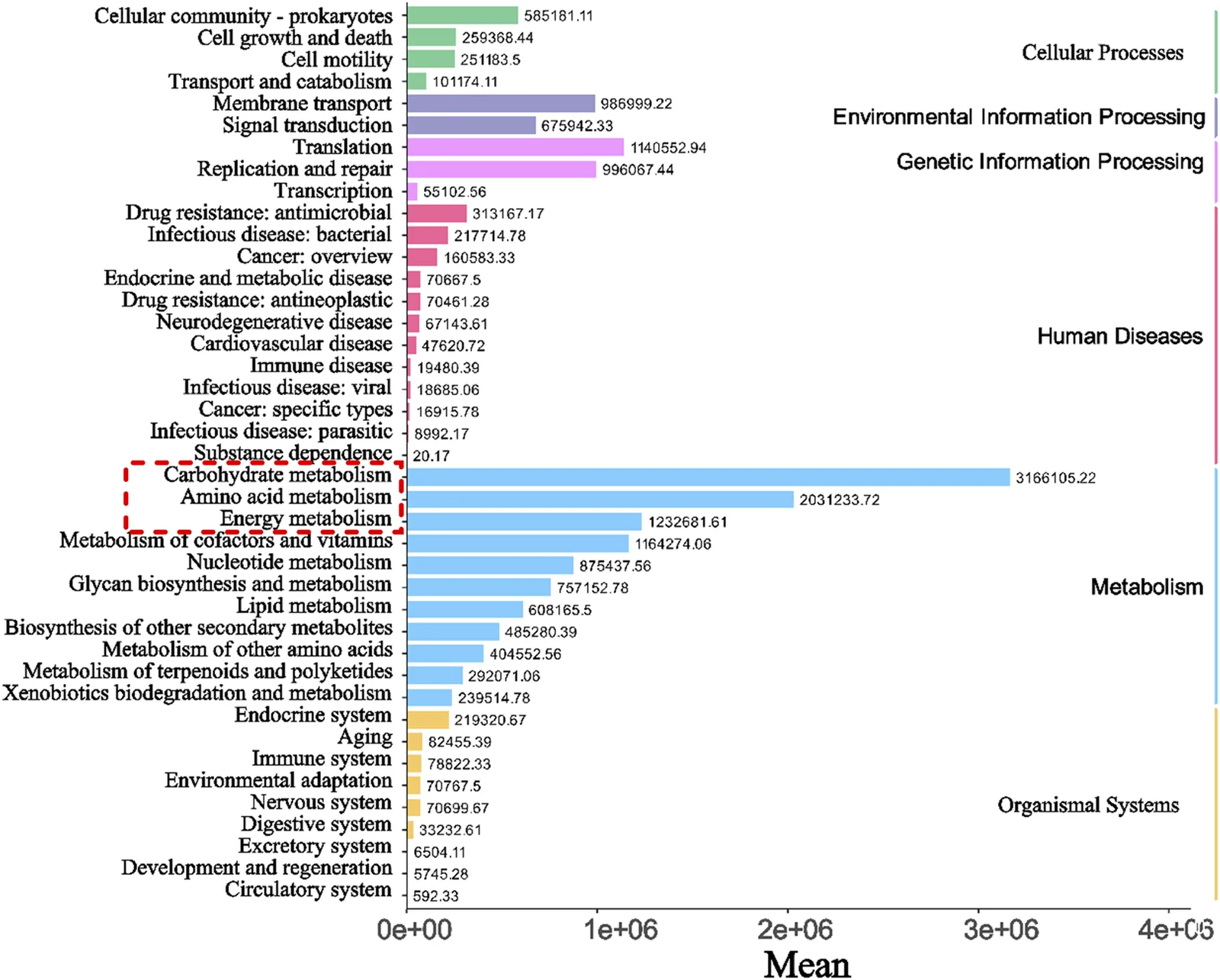
Functional profile of gut microbial communities based on KEGG pathway annotation. The bar plot shows the mean abundance of microbial gene families annotated to KEGG pathways, categorized into higher-level functional modules (color-coded). The x-axis represents the mean abundance of functional categories across all samples, with longer bars indicating higher functional gene enrichment. (n = 6 for each group).

To investigate the functional implications of APS-induced gut microbiota remodeling, we performed KEGG pathway annotation of microbial gene families. As shown in Figure 5, the most highly enriched functional pathways were associated with metabolism, particularly Carbohydrate metabolism (highest mean abundance, 3.16 × 10^6^), Amino acid metabolism (2.01 × 10^6^), and Energy metabolism (∼1.23 × 10^6^). These pathways are critical for maintaining host metabolic homeostasis and producing microbial metabolites (e.g., short-chain fatty acids) that modulate the gut-brain axis.

### 3.6 APS regulates fecal metabolite profiles and their correlations with gut microbiota in 5×FAD mice

To characterize the metabolic consequences of gut microbiota remodeling by AP S, we performed targeted metabolomic profiling of fecal samples and integrated these data with microbial taxonomic profiles.

**FIGURE 6:**
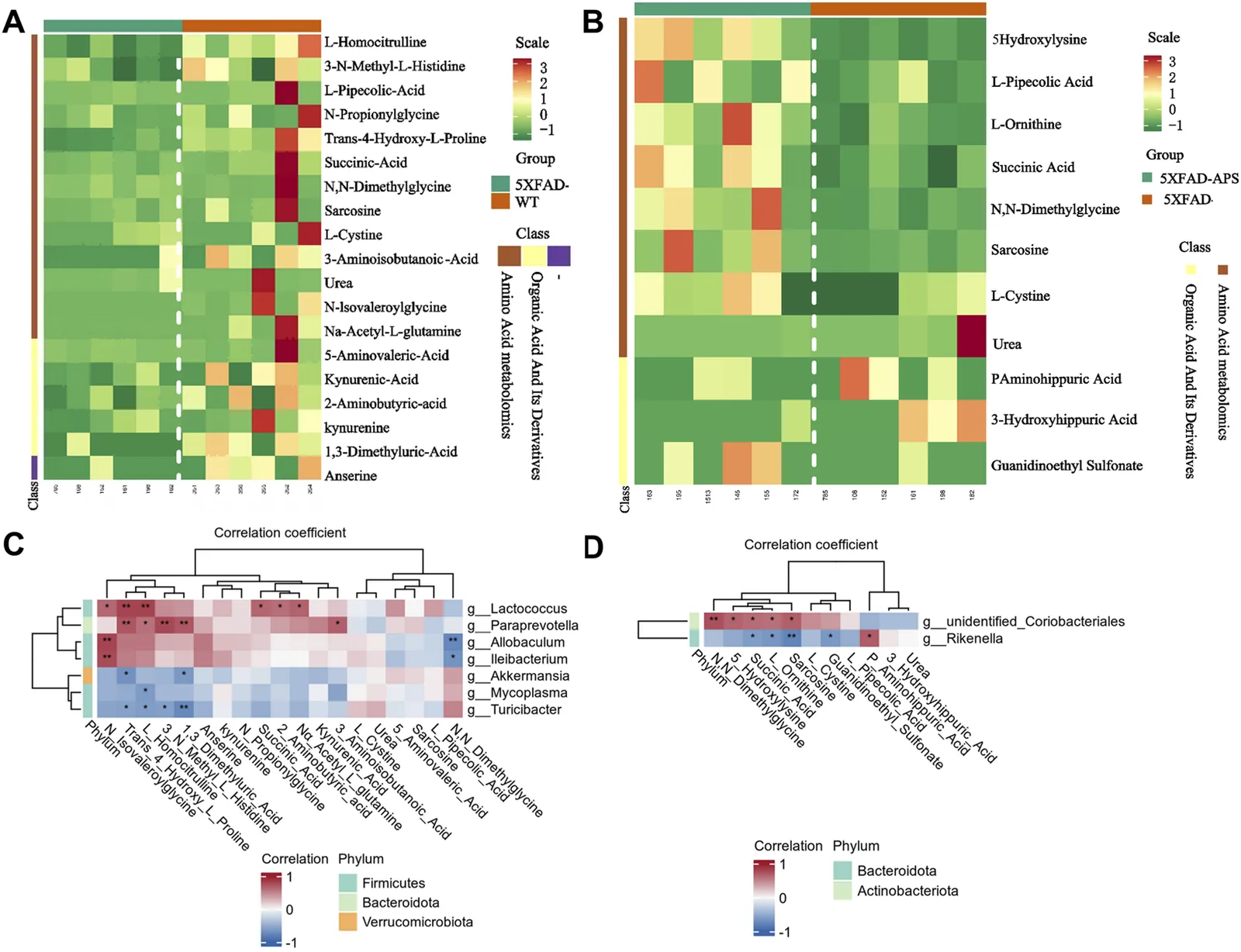
Metabolomic profiling and microbiota-metabolite correlations in response to APS intervention. **(A)** Heatmap of organic acid and derivative metabolites in wild-type (WT) and 5×FAD mice. Color intensity represents normalized metabolite abundance (red: high, green: low). **(B)** Heatmap of amino acid and derivative metabolites in 5×FAD and 5×FAD + APS (APS-treated) mice. Color intensity represents normalized metabolite abundance (red: high, green: low). **(C)** Correlation heatmap between gut microbiota (genus level) and differential metabolites in the WT vs. 5×FAD comparison. Red indicates positive correlation; Blue indicates negative correlation. Phylum-level taxonomic affiliations are annotated on the right. **(D)** Correlation heatmap between gut microbiota (genus level) and differential metabolites in the 5×FAD vs. 5×FAD + APS comparison. Red indicates positive correlation; blue indicates negative correlation. Phylum-level taxonomic affiliations are annotated on the right. Correlation significance was determined by Spearman’s correlation, with *p < 0.05 (significant) and **p < 0.01 (highly significant). correlation test, with *p < 0.05 and **p < 0.01. (n = 6 for each group).

As shown in Figure 6A, the 5×FAD group exhibited significant perturbation in organic acid metabolism, with decreased levels of N,N-Dimethylglycine, Succinic-Acid and Sarcosine relative to the WT group. Conversely, APS intervention (Figure 6B) reversed these changes, restoring levels of N,N-Dimethylglycine, Succinic acid, and L-cystine toward WT baseline, while reducing the abundance of pro-inflammatory metabolites such as p-aminohippuric acid and 5-hydroxykynurenic acid.

Correlation analysis revealed strong associations between microbial taxa and differential metabolites (Figures 6C,D). In the WT vs. 5×FAD comparison (Figure 6C), the depletion of commensal genera Lactococcus and Allobaculum (phylum Firmicutes) positively correlated with elevated Succinic-Acid, 2-Aminobutyric-acid, Trans-4-Hydroxy-L-Proline, N,N-Dimethylglycine. In the 5×FAD vs. 5×FAD + APS comparison (Figure 6D), APS-induced enrichment of Rikenella and unidentified Coriobacteriales (phylum Actinobacteriota) correlated with the levels of L-cysteine, Sarcosine, and Succinic-Acid.

Together, these findings demonstrate that APS intervention remodels both the gut microbiota and its metabolic output, shifting the metabolic profile from a pro-inflammatory, AD-associated state toward a homeostatic, neuroprotective phenotype. The observed microbiota-metabolite correlations further support the role of the gut-brain axis in mediating APS’s effects on cognitive function.

### 3.7 Fecal microbiota transplantation (FMT) of APS-modulated gut microbiota improves cognitive function in 5×FAD mice

**FIGURE 7:**
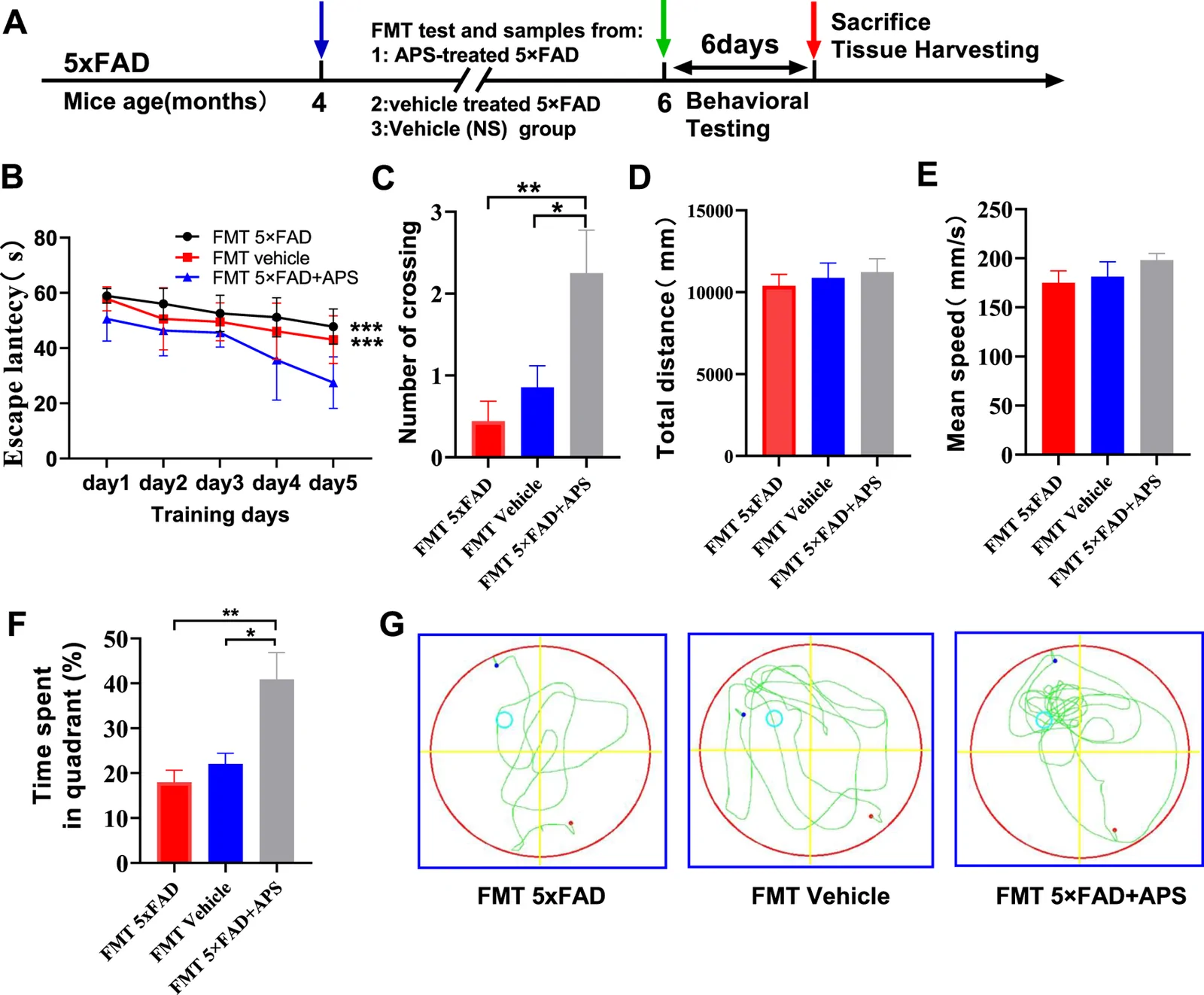
Fecal microbiota transplantation (FMT) of APS-modulated gut microbiota improves cognitive function in 5×FAD mice. **(A)** Timeline of the FMT experiment: 4-month-old 5×FAD mice received FMT from APS-treated/vehicle-treated 5×FAD donors (or vehicle control), followed by 6 days of behavioral testing prior to sacrifice. **(B–H)** Morris water maze (MWM) performance in FMT groups: **(B)** Escape latency during the 5-day training phase; **(C)** Number of platform crossings; **(D)** Total swimming distance; **(E)** Mean swimming speed; **(F)** Percentage of time spent in the target quadrant. **(G)** Representative swimming trajectories during the probe trial. Data are presented as mean ± SEM (n = 8 per group). *p < 0.05, **p < 0.01, ***p < 0.001. Only significant differences are marked, and no symbols are added for comparisons without statistical significance.

To confirm that APS exerts cognitive benefits via gut microbiota modulation, we performed fecal microbiota transplantation (FMT) (Figure 7A): 5×FAD mice received microbiota from APS-treated 5×FAD donors (FMT 5×FAD + APS), vehicle-treated 5×FAD donors (FMT 5×FAD), or FMT Vehicle (oral gavage of sterile normal saline). MWM testing revealed that FMT 5×FAD + APS mice exhibited improved spatial memory relative to FMT 5×FAD controls: During 5 days of training (Figure 7B), the FMT 5×FAD + APS group exhibited a progressive reduction in escape latency, compared to the FMT 5×FAD control group and FMT Vehicle group (*p* < 0.001). In the probe trial, mice receiving APS-modulated microbiota crossed the target platform location significantly more times (Figure 7C, *p* < 0.01) and spent a greater percentage of time in the target quadrant (Figure 7F, *p* < 0.01) compared with FMT 5×FAD,and *p* < 0.05 compared with FMT Vehicle, indicating enhanced spatial memory retention, while total swimming distance and mean speed (Figures 7D,E) were unchanged—ruling out locomotor differences as a confounding factor. Swimming trajectories (Figure 7G) further reflected targeted exploration of the platform area in FMT 5×FAD + APS mice, compared to random swimming in FMT 5×FAD controls. These data confirm that APS-modulated gut microbiota directly contribute to cognitive improvement in 5×FAD mice.

### 3.8 Fecal microbiota transplantation (FMT) of APS‐modulated gut microbiota attenuates microglial activation and reshapes neuroinflammation in 5×FAD mice

**FIGURE 8:**
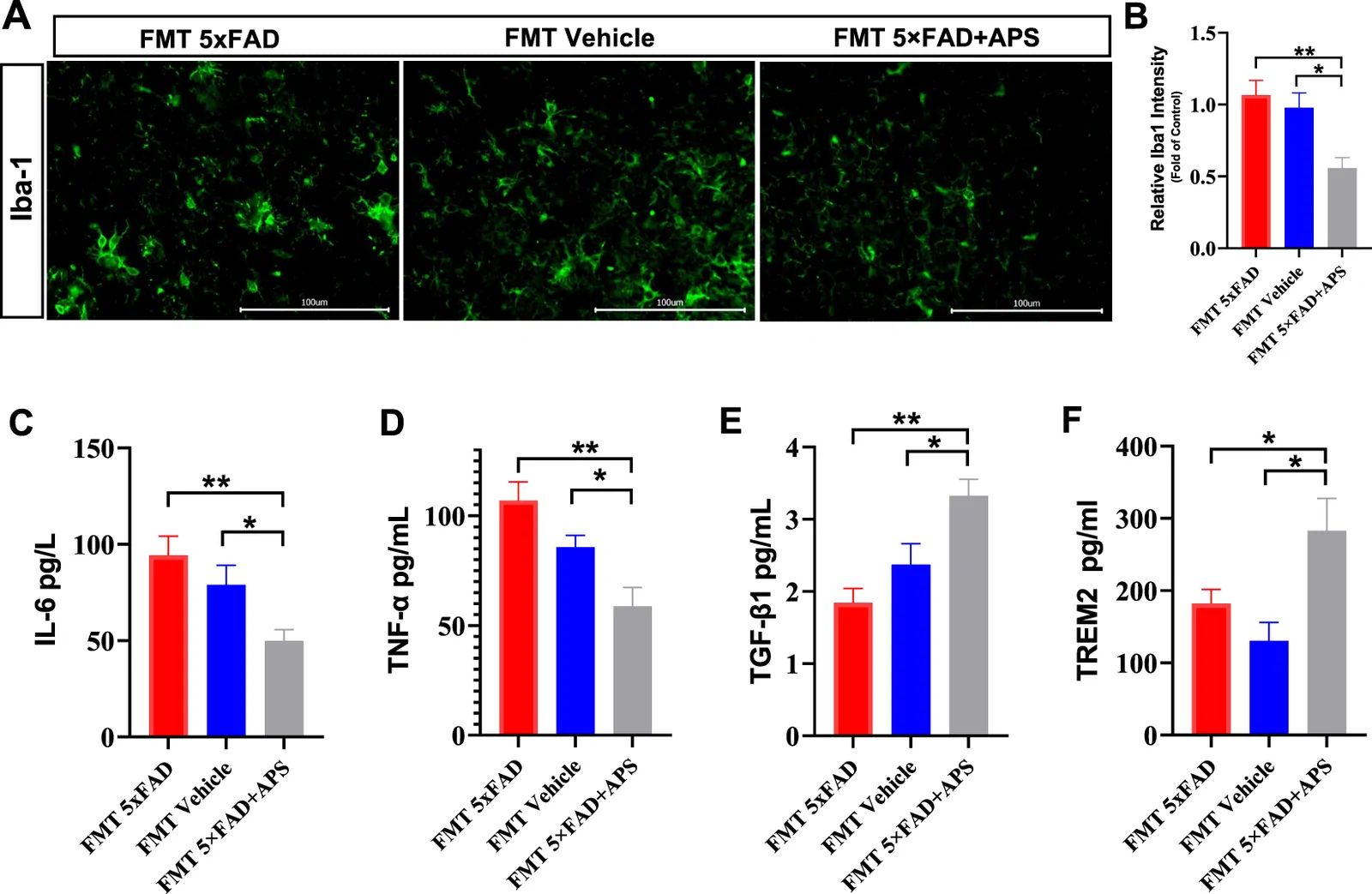
FMT of APS-modulated microbiota attenuates microglial activation and reshapes neuroinflammation in 5×FAD mice. **(A)** Representative immunofluorescence images of Iba-1 (microglial marker) in the cortex; scale bar = 100 μm. **(B)** Quantitative analysis of relative Iba-1 fluorescence intensity. **(C–F)** Levels of inflammatory cytokines in hippocampal tissues: **(C)** IL-6; **(D)** TNF-α; **(E)** TGF-β1; **(F)** TREM2. Data are presented as mean ± SEM. (n = 6 in A,and n = 5-6 in **(C–F)** *p < 0.0 5, **p < 0.01, ***p < 0.001.

To confirm that APS-modulated gut microbiota drive the anti-neuroinflammatory effects of APS, we assessed microglial activation and inflammatory mediators in FMT groups. As shown in Figures 8A,B, FMT 5×FAD mice (receiving 5×FAD-donor microbiota) exhibited robust microglial activation, marked by enlarged cell bodies, thickened and truncated processes, and a clustered spatial distribution; in contrast, FMT 5×FAD + APS mice (receiving APS-donor microbiota) showed a significant reduction in Iba-1 intensity. Consistent with this, FMT 5×FAD + APS mice displayed decreased levels of pro-inflammatory cytokines IL-6 (Figure 8C; *p* < 0.01 vs. FMT 5×FAD and *p* < 0.05 vs. FMT vehicle) and TNF-α (Figure 8D; *p* < 0.01 vs. FMT 5×FAD,and p < 0.05 vs. FMT vehicle), alongside increased anti-inflammatory TGF-β1 (Figure 8E; *p* < 0.01 vs. FMT 5×FAD,and *p* < 0.05 vs. FMT vehicle) and microglial phagocytosis-related TREM2 (Figure 8F; *p* < 0.05 vs. FMT 5×FAD or vs. FMT vehicle). FMT Vehicle mice showed no significant changes in these indices (vs. FMT 5×FAD). These data confirm that APS-modulated gut microbiota mediate the suppression of microglial overactivation and restoration of a homeostatic inflammatory profile in 5×FAD mice.

**FIGURE 9:**
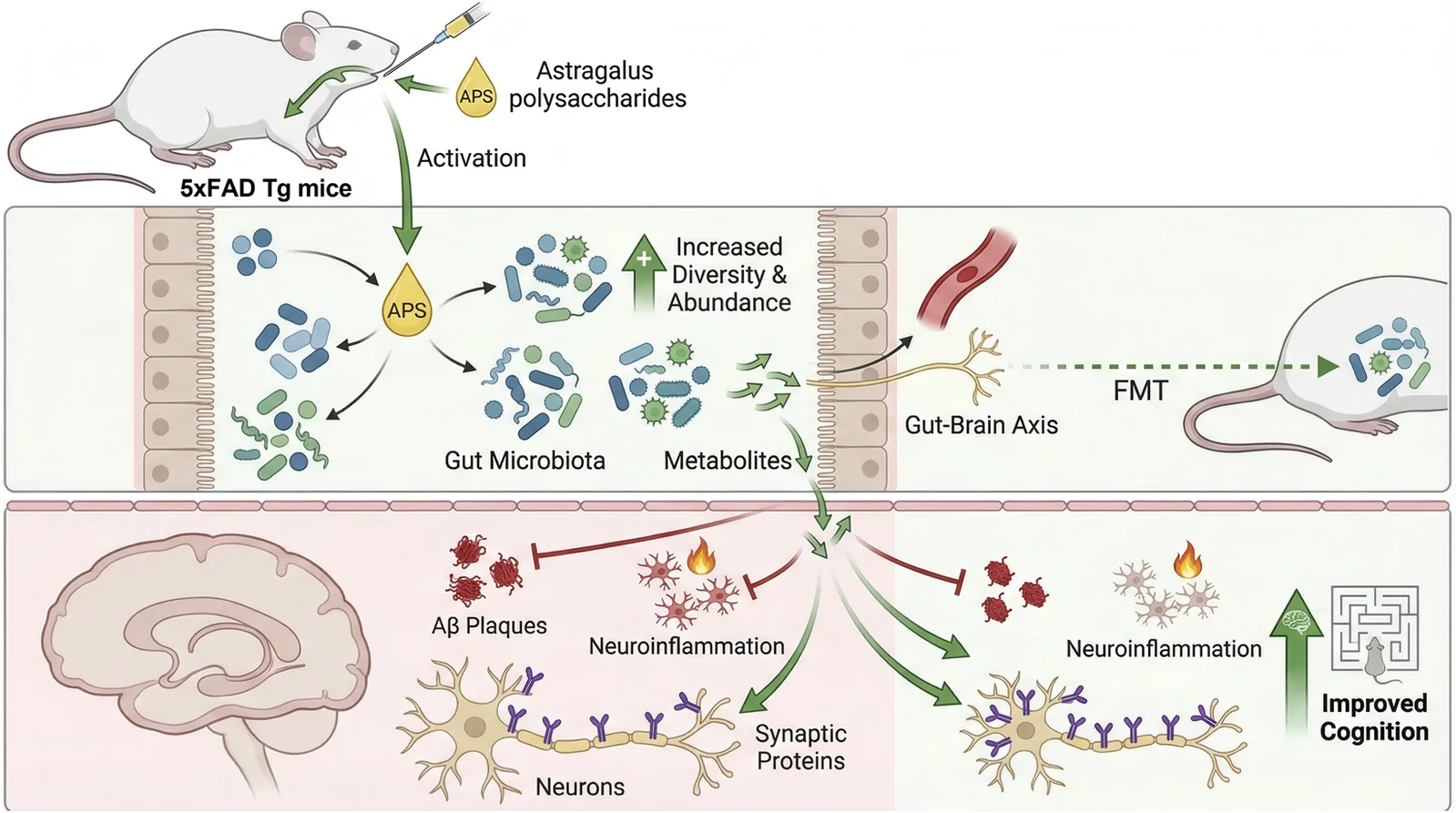
Main schematic overview and Graphical abstract in this study.

Collectively, all key experimental designs, phenotypic observations and core molecular findings of the present study are integrated and summarized in Figure 9, which serves as the main schematic overview and graphical abstract of this work.

## 4 Discussion

Alzheimer’s disease (AD) is a multifactorial neurodegenerative disorder. Growing evidence highlights the gut-brain axis as a key modulator of AD pathogenesis, suggesting that gut microbiota and gut-brain axis (GBA) play a pivotal role in regulating neuroinflammation and cognitive function (Dissanayaka et al., 2024; Giridharan et al., 2022). In addition, AD is characterized by significant gender differences in risk and mechanisms, including the influence of gender on the regulation of intestinal flora and on therapeutic response to medicine. To enhance experimental consistency and minimize the confounding influence of sex differences (Bosch et al., 2024), particularly the effect of estrogen (Bostick et al., 2025), we chose to use male mice in this study. Natural products derived from traditional medicine have attracted considerable attention for their multi-target therapeutic potential and favorable safety profile. Among these, natural polysaccharides, are especially promising owing to their low toxicity and broad pharmacological actions. *Astragalus polysaccharide* (APS)-a bioactive polysaccharide from *Astragalus membranaceus* composed of glucose, arabinose, galactose, and rhamnose-has shown notable utility in central nervous system diseases (Shi and Ma, 2024).

In this study, male 5×FAD mice, which are widely used and well-characterized, received APS at a dose of 200 mg/kg/day via oral gavage for 60 consecutive days and underwent behavioral and neuropathological analyses, as well as assessments of gut microbiota and metabolism. We demonstrated that both direct gavage administration and fecal microbiota transplantation (FMT) from APS-treated donors improved spatial memory and learning ability in behavioral testing. Our study revealed that APS administration significantly reduced amyloid-β plaque deposition, enhanced expression of memory-related presynaptic protein markers such as syntaxin (Syn) and postsynaptic density protein (PSD-95), and attenuated neuroinflammatory cytokines. Notably, APS treatment markedly readjusted the abundance, diversity, and metabolite output of the gut microbiota in 5×FAD mice. Furthermore, FMT from APS-treated donors confirmed the involvement of the gut–brain axis and the protective effects in attenuating neuroinflammation mediated by microglial activation.

Of particular note, APS-treated mice exhibited levels of anti-inflammatory cytokine TGF-β1 and microglial phagocytosis-related molecule TREM2 (Figures 2G,H) that were not only significantly elevated compared to untreated 5×FAD mice, but also surpassed those observed in wild-type (WT) controls. Similarly, the postsynaptic density protein PSD-95 (Figures 3D,E) was restored beyond WT baseline levels following APS treatment. These findings may suggest an overcompensatory anti-inflammatory and synaptic recovery response induced by APS, possibly reflecting a rebound effect following prolonged Aβ-driven suppression of homeostatic microglial function and synaptic signaling. Alternatively, this supra-physiological elevation could indicate that APS actively drives a pro-reparative state rather than merely restoring basal homeostasis. While the precise mechanisms underlying this above-normal induction remain to be elucidated, these observations underscore the robust neuromodulatory and immunomodulatory capacity of APS and warrant further investigation into optimal dosing strategies.

Consistent with its established role in Alzheimer’s disease (AD) pathogenesis, microglia—the primary immune cells of the central nervous system—undergo detrimental activation in response to Aβ, leading to the secretion of pro-inflammatory cytokines that drive neuronal dysfunction (Chen and Holtzman, 2022). Similarly, our data showed that global microglial activation is hallmark in 5×FAD mice (Wei et al., 2025). In the present study using 5×FAD mice, we found that APS treatment effectively suppressed this pathological microglial activation, as evidenced by reduced Iba-1 expression. Concurrently, APS shifted the cerebral inflammatory milieu toward homeostasis, characterized by the downregulation of pro-inflammatory cytokines IL-6 and TNF-α and the upregulation of anti-inflammatory TGF-β1 and phagocytosis-related (TREM2) factors. This rebalancing of the inflammatory profile underscores the capacity of APS to ameliorate AD-related pathology, at least in part, through the modulation of microglia-mediated neuroinflammation. The therapeutic strategy of favoring a protective, anti-inflammatory (M2) over a detrimental, pro-inflammatory (M1) microglial phenotype is well-supported in the field (Darwish et al., 2023; Si et al., 2023), with M2 microglia being instrumental in post-damage repair (McManus et al., 2025). The FMT study further provides bidirectional evidence for APS-induced modulation of microglial polarization. Converging evidence underscores the therapeutic value of APS in countering Aβ pathology in AD. Our findings align with prior research on Astragalus-derived compounds and extend these observations, demonstrating that APS treatment attenuates Aβ-driven pathogenesis (Qin et al., 2020). Similarly, it has been demonstrated that APS exerts apparent neuroprotection via anti-inflammatory actions and immune cell regulation in a pentylenetetrazole-induced kindling mouse model (Lu et al., 2024) and experimental autoimmune encephalomyelitis (Zhao et al., 2024).

A key advance from our analysis is the finding that APS upregulated the expression of synapse-associated proteins Syn and PSD95, and markedly improved behavioral measures of cognitive deficits in APS-treated mice. It is widely known that synapses play a crucial role in neuronal communication (Parra-Damas and Saura, 2019). Any abnormality or structural deformation due to endogenous or exogenous stress may lead to neurological conditions (Wilkinson et al., 2024), especially in AD (Zhao et al., 2020). Synaptic proteins are responsible for the exocytosis and endocytosis of various neurotransmitters and have been found to be altered in neurodegenerative disorders (Bhembre et al., 2023). Synaptic loss and dysfunction, including the downregulation of pre- and postsynaptic proteins such as PSD-95 and synaptosomal-associated protein 23 (SNAP-23), are central features of AD and are evident in 5×FAD mice (Zeng et al., 2015). Previous studies have reported that the amelioration of neuroinflammation is critically linked to synaptic integrity, a key determinant of cognitive function (Ebrahimi et al., 2025). Building upon the established knowledge that Aβ pathology impairs synaptic protein expression (Yu et al., 2024), our data reveal a significant restorative effect of APS. Specifically, APS treatment counteracted the Aβ-induced decrease in key synaptic markers, notably upregulating the expression of the postsynaptic density protein PSD-95. This correction of synaptic molecular deficits was paralleled by robust improvements in cognitive behavior. APS-treated 5×FAD mice exhibited significantly reduced escape latency during the acquisition phase of the Morris water maze (MWM) and, in the probe test, spent more time in the target quadrant and made more platform crossings, indicating enhanced spatial learning and memory. In summary, our results demonstrate that APS alleviates cognitive deficits in 5×FAD mice, and this effect is associated with the upregulation of synaptic proteins such as PSD-95. We propose that this synaptic protection is a downstream consequence of APS-mediated mitigation of Aβ-driven neuroinflammation through modulation of microglial activation and polarization.

Notably, APS treatment elevated TGF-β1, TREM2, PSD-95 and Syntaxin levels beyond not only 5×FAD mice but also wild-type controls. This supra-physiological upregulation reflects a compensatory neuroprotective effect of APS in AD pathological microenvironment. Increased TGF-β1 further suppresses neuroinflammatory cascade and maintains immune homeostasis (Bedolla et al., 2024). Upregulated TREM2 facilitates microglial phenotypic transformation and enhances Aβ phagocytic clearance (Wei et al., 2025). Meanwhile, the recovery of synaptic proteins exceeding basal level indicates that APS not only rescues AD-induced synaptic loss but also improves synaptic plasticity. Nevertheless, the current conclusion is limited by sample size; future studies with enlarged samples will further verify the regulatory mechanism of APS on these supra-physiological molecular expressions.

Accordingly, establishing a functional link between gut microbiota modulation brain inflammation (including synaptic integrity), and ultimate cognitive function is of particular importance in AD research (Kapoor et al., 2024; Lei et al., 2025). Previous studies have demonstrated a strong association between gut dysbiosis and neuroinflammatory dysregulation in AD pathogenesis (Luo et al., 2022). Although APS has been linked to anti-inflammatory effects, alterations in gut microbiota, and cognitive improvement in diabetic mouse models, the mechanisms involved require further clarification in AD (Liu et al., 2019). In alignment with previous reports, our study observed restoration of alpha diversity indices in APS-treated mice, indicating a partial recovery of microbial community richness and evenness, which are typically compromised in this AD model (Hong et al., 2020). More profoundly, beta diversity analyses (PCoA and NMDS) revealed that APS treatment shifted the overall microbial community structure of 5×FAD mice away from the diseased state and toward a configuration more closely resembling that of wild-type controls (Yun et al., 2023). This taxonomic remodeling was driven by specific alterations in signature taxa, including a reduction in the relative abundance of certain *Lactobacillus* species (e.g., L. johnsonii and L. reuteri) that were enriched in 5×FAD model. These findings are consistent with the growing recognition of gut microbiota as a modifiable target in AD and suggest that APS can counteract AD-associated dysbiosis (Liu et al., 2019; Wang et al., 2019).

To further and systematically evaluate the therapeutic potential and underlying mechanisms of APS in 5×FAD transgenic mice, we performed microbial functional analysis via KEGG pathway analysis (Figure 5). Relative to WT controls, the 5×FAD model exhibited a skewed functional profile: disease-associated pathways, especially carbohydrate metabolism, amino acid metabolism and energy metabolism. These pathways are critical for maintaining host metabolic homeostasis and producing microbial metabolites (Sun et al., 2022). Subsequently, targeted metabolomics was performed to systematically link microbial dysbiosis to host metabolic disturbances, with a particular focus on SCFAs and related metabolites. As shown in Figure 6, the 5×FAD mice displayed marked perturbations in organic acid metabolism, including decreased levels of Succinic-Acid, sarcosine,N,N-Dimethylglycine, and 2-Aminobutyric-acid-metabolites associated with energy, oxidative stress and neuroinflammation (Gallart-Palau et al., 2017; Lendvai et al., 2023; Peruzzotti-Jametti et al., 2018), which were reversed by APS intervention. Meanwhile, APS restored the levels of protective metabolites such as L-pipecolic acid and succinic acid and reduced the abundance of excitotoxicity metabolites, including p-aminohippuric acid, which have been linked to glutamate-mediated synaptic dysfunction (Weber et al., 2001). Notably, metabolomic profiling revealed an enrichment of key amino acid-related metabolites, including succinic acid and N,N-dimethylglycine, in APS-treated AD mice with reduced amyloid pathology, supporting a potential preventive role through modulation of neurotransmitters, their precursors, and energy metabolism (Kim et al., 2025; Niu et al., 2025). Furthermore, APS regulated several SCFA-related pathways, particularly those involving 2-aminobutyric acid and 5-aminolevulinic acid, whose downstream products have been implicated in anti-inflammatory effects along the gut–brain axis in 5×FAD mice, consistent with findings from other disease models such as chronic fatigue syndrome (Wei et al., 2023).

Collectively, the restoration of microbial diversity, normalization of community structure, and correction of functional pathway imbalances constitute a multifaceted mechanism by which APS modulates the gut ecosystem. This remodeling of the gut microbiota is proposed to represent a fundamental upstream step in the therapeutic mechanism of APS, with downstream influences on host physiology—including systemic inflammation and neuroinflammation—mediated via the gut-brain axis (Yue et al., 2023). The subsequent analysis of neuroinflammatory markers and cognitive outcomes in our study were designed to trace these causal links, supporting the hypothesis APS-induced normalization of gut microbial taxonomy and function contributes to the alleviation of central nervous system pathology in AD (Eicher and Mohajeri, 2022). Of course,the specific molecular mechanisms by which amino acids-related metabolites affect cognition remain incompletely understood, underlining the need for a deeper characterization of the microbiota-gut-brain axis (Eicher and Mohajeri, 2022). To our knowledge, this is the first report demonstrating that APS administration significantly remodels the dysbiotic gut microbiota in a mouse model of Alzheimer’s disease. Furthermore, from a nutritional perspective, APS is abundant in bioactive polysaccharides and antioxidant compounds, contributing to enhanced immune function and delayed cognitive decline (Shi and Ma, 2024). Notably, the regulatory effects of APS on immunity, gut microbiota, and metabolic pathways are not limited to Alzheimer’s disease but have also been observed in diabetic murine models (Liu et al., 2019), chronic fatigue syndrome (Wei et al., 2023), ulcerative colitis in high-fat diet-induced obese mice (Yue et al., 2023), and bleomycin-induced pulmonary fibrosis (Wei et al., 2023). These multifaceted benefits underscore the broad therapeutic potential and natural value of APS within the framework of traditional Chinese medicine for nutritional health and disease prevention (Liu et al., 2025; Shi and Ma, 2024).

## 5 Limitations and future directions

While this investigation provides comprehensive evidence for APS-mediated neuroprotection via the gut–brain axis, several limitations should be acknowledged. First, while we identified broad structural changes in microbial communities, the specific bacterial taxa and molecular mechanisms underlying the cognitive benefits remain to be fully elucidated. Further studies are needed to clarify whether AD patients exhibit similar microbiota alterations linked to cognitive decline. Second, the signaling pathways through which gut microbiota modulate microglial polarization require deeper exploration. Third, the use of a single transgenic mouse model limits the generalizability of our findings; future studies should validate these results in other AD models, such as tauopathy models (e.g., PS19), and ultimately in human cohorts, where correlations between gut microbiota and cognition have been reported (Zhou et al., 2021). Fourth, the optimal dosage and treatment duration of APS remain undetermined and warrant systematic pharmacokinetic and pharmacodynamic studies. Additionally, while the involvement of microglia has been emphasized, the potential role of astroglia in APS-mediated regulation of neuroinflammation should not be overlooked. These limitations underscore the necessity for future studies focusing on the aforementioned issues to further clarify the mechanistic underpinnings and therapeutic potential of APS. Future research directions should also encompass the further exploration and innovation of therapeutic strategies and delivery systems, such as the prevention or treatment of Alzheimer’s disease (AD) via the administration of APS-containing capsules or chewable tablets as part of daily dietary supplements, analogous to the supplementation of dietary fiber (Zhao et al., 2025).

## 6 Conclusion

This preclinical investigation demonstrates that APS exhibits potential in alleviating amyloid-β-induced pathological changes, primarily through its anti-neuroinflammatory effects. The observed neuroprotection appears to involve multiple mechanisms, including regulation of the gut-brain communication pathway and suppression of hippocampal microglial activation. Although these findings provide initial evidence supporting APS’s neuroprotective capacity, further clinical studies are required to validate its therapeutic potential in human subjects.

## Data Availability

The data presented in the study are deposited in the figshare repository, https://doi.org/10.6084/m9.figshare.33032705.
